# Ultrasound and Scintigraphy for Preoperative Localization in Primary Hyperparathyroidism: A Single-Center Experience

**DOI:** 10.7759/cureus.100488

**Published:** 2025-12-31

**Authors:** Olorunleke M Arokoyo, Graham Garside, Lauren Bolton, Frank Agada

**Affiliations:** 1 Otolaryngology, York and Scarborough Teaching Hospitals NHS Foundation Trust, York, GBR

**Keywords:** parathyroidectomy, parathyroid scintigraphy, primary hyperparathyroidism, sestamibi, ultrasound

## Abstract

Introduction

Primary hyperparathyroidism is an endocrine condition characterized by elevated parathyroid hormone, often with high calcium levels, though calcium may remain normal in some cases. The condition is most frequently caused by a single parathyroid adenoma. Surgical removal of the affected gland is generally recommended for symptomatic patients, and modern surgical approaches, including minimally invasive techniques, are guided by preoperative imaging. Ultrasound (US) is commonly used, and an additional scan, such as a sestamibi scan, may be employed to aid surgical planning. This study reviewed cases of parathyroid surgery over nine years at a single center to evaluate the utility of US and sestamibi parathyroid scintigraphy (PS) for preoperative localization.

Methods

In this retrospective study, we examined cases of parathyroid surgery for primary hyperparathyroidism at the Department of Otolaryngology, York District Hospital, York, United Kingdom, over a nine-year period. A total of 316 patients underwent parathyroid surgery during the study period. Nineteen cases were excluded: 17 had tertiary hyperparathyroidism, and two had a preoperative diagnosis of multiple endocrine neoplasia type 1 and underwent four-gland excision. The remaining 297 cases, in which a likely candidate lesion for primary hyperparathyroidism was identified prior to surgery, were included.

Results

The average age was 61.3 ± 12.7 years, with 235 (78.6%) females. US demonstrated a sensitivity of 79.4%, a specificity of 64.3%, a positive predictive value (PPV) of 95.3%, and a negative predictive value (NPV) of 25.7%. Scintigraphy had a sensitivity of 76.2%, a specificity of 48.3%, a PPV of 91.6%, and an NPV of 21.5%. Combined US and scintigraphy showed a sensitivity of 59.4%, specificity of 77.8%, PPV of 95.5%, and NPV of 19.6%. The OR of cure for revision versus first operation was 0.20 (p = 0.001, 95% CI: 0.072-0.528). The OR of cure in positive versus negative US cases, controlling for scintigraphy status, was 5.233 (95% CI: 2.14-12.775, p < 0.0001). Scintigraphy alone was not a significant predictor of cure (OR 2.169, 95% CI: 0.904-5.204, p = 0.083). In an analysis of US as a predictor of cure, controlling for scintigraphy status and whether the operation was a revision or first operation, only US status remained a significant predictor (OR 4.47, p = 0.001, 95% CI: 1.77-11.251).

Conclusions

Image-guided focused parathyroidectomy results in superior cure rates in patients with primary hyperparathyroidism. In our practice, US predicted a high rate of all-cause cure and cure when histology corresponded with US findings. We were unable to show that PS, as an adjunct to US, significantly improved cure rates. Patient groups at high risk of operative failure include those undergoing reoperation, those without positive US or scintigraphy findings, and those with concomitant thyroid pathology. In these patients, further imaging, such as 4D CT, could be considered to delineate candidate lesions in greater detail for preoperative planning.

## Introduction

Primary hyperparathyroidism is a common endocrine disorder defined by abnormally elevated levels of parathyroid hormone (PTH), typically associated with hypercalcemia [1], or, in some cases, normocalcemia with no identifiable cause for elevated PTH levels [2]. It is the leading cause of hypercalcemia in the community [3], with a prevalence of 0.84% and an incidence of four to six per 10,000 person-years; the incidence is approximately two and a half times higher in women than in men [4]. Clinical manifestations of primary hyperparathyroidism can range from completely asymptomatic to severe and disabling [5]. Symptoms are often non-specific and varied, including weakness and fatigue, anxiety, cognitive impairment, nephrolithiasis, constipation, reduced bone density leading to pathologic fractures, and muscle aches, among others. Solitary adenomas (85%) are the most common cause of primary hyperparathyroidism, with hyperplasia, double adenomas, and, rarely, parathyroid carcinomas accounting for other cases [6].

Parathyroidectomy is the recommended treatment for symptomatic patients, with contemporary surgical approaches, including minimally invasive surgery, facilitated by preoperative imaging. Preoperative imaging has been shown to reduce treatment burden, including complications such as permanent hypoparathyroidism, while improving recovery time and reducing costs [7].

Ultrasound (US) and parathyroid scintigraphy (PS) are widely used to localize candidate lesions preoperatively, either as a single modality or in combination. US uses high-frequency sound waves from a transducer to generate images of the examined area. Scintigraphy relies on the uptake of radioactive tracers in metabolically active cells, such as parathyroid adenomas, to highlight those areas. A systematic review and meta-analysis of 12 studies found no significant differences in sensitivity between the two modalities (pooled sensitivity: 80% for US, 84% for PS; pooled specificity: 77% for US, 87% for PS). Individual study outcomes were heterogeneous and conflicting, likely due to the operator-dependent nature of US, variability in the interpretation of PS, and small sample sizes. NICE guidelines recommend offering preoperative imaging, usually US, before parathyroidectomy to guide the surgical approach. A second form of preoperative imaging, typically a sestamibi scan, is recommended if further guidance for surgical planning is needed [8].

The aim of this retrospective study was to investigate the utility of US and PS in preoperative localization for patients with primary hyperparathyroidism. The primary objective was to describe the sensitivity, specificity, positive predictive value (PPV), and negative predictive value (NPV) of US, PS, and combined US and PS. The secondary objective was to analyze positive US and positive PS as predictors of cure.

This article was previously presented as an oral presentation at the 6th Edition of the Global Conference on Surgery and Anaesthesia on September 16, 2025.

## Materials and methods

In this retrospective study, we examined cases of parathyroid surgery for primary hyperparathyroidism at the Department of Otolaryngology, York District Hospital, York, United Kingdom, over a nine-year period (January 2012 to 2021). Primary hyperparathyroidism was diagnosed, assessed, and managed according to NICE guidelines [8]. Electronic patient records were reviewed for relevant data, including demographics; pre-, intra-, and postoperative serum PTH and adjusted serum calcium; imaging reports for US and sestamibi PS; operative approach classified as unilateral operation or four-gland exploration; and histology.

Inclusion criteria included patients who underwent parathyroidectomy for primary hyperparathyroidism and were subsequently diagnosed with adenoma, hyperplasia, or carcinoma, as well as reoperations with subsequent repeat imaging. Exclusion criteria included cases where histology, imaging, or follow-up data were unavailable and patients with tertiary hyperparathyroidism or multiple endocrine neoplasia type 1 (MEN 1). Cases were selected by simple random sampling. A total of 316 patients underwent parathyroid surgery during the study period. Nineteen cases were excluded: 17 had tertiary hyperparathyroidism, and two had a preoperative diagnosis of MEN 1 and underwent four-gland excision. The remaining 297 cases, in which a likely candidate lesion for primary hyperparathyroidism was identified preoperatively, were included.

US scans were performed using a Philips EPIQ system (Philips Healthcare, Amsterdam, Netherlands) with an eL 18-4 transducer. For PS, patients underwent dual-phase technetium-99m sestamibi with planar and single-photon emission CT (SPECT) imaging, with a few cases also having CT. All US and PS images were reviewed and reported by head and neck radiologists experienced in identifying candidate lesions for parathyroid surgery. Imaging findings were considered positive when a candidate lesion was identified and confirmed by surgical and histological findings. Concordance was defined as the detection of positive ipsilateral findings on both imaging modalities.

Surgery was performed almost exclusively by a single surgeon. Preoperative planning determined whether to undertake a focused parathyroidectomy or four-gland exploration, based on preoperative imaging in accordance with NICE guidelines [8]. Parathyroidectomy was performed via a conventional approach, using a harmonic scalpel for dissection and bipolar diathermy for hemostasis. Focused parathyroidectomy was performed when the lesion was identified and localized with concordant imaging, while four-gland exploration was performed in cases where the gland was not localized preoperatively, the patient had multi-gland disease, or imaging results were discordant. In cases where candidate lesions were not identified macroscopically, focused procedures were extended to four-gland exploration; this was included in the preoperative discussion and consenting process.

Patients were considered cured following histological confirmation and biochemical normalization of adjusted serum calcium (2.2-2.6 mmol/L) and PTH (1.8-7.9 pmol/L) postoperatively. Follow-up occurred in the ENT clinic at six weeks post-surgery and subsequently at six months and one year postoperatively in the endocrinology department, which could refer patients back to ENT if concerns arose or discharge them if stable.

Statistical analysis was performed using IBM SPSS Statistics for Windows, Version 27.0 (Released 2019; IBM Corp., Armonk, NY, USA). Sensitivities of US, PS, concordant US and PS, and positive US and/or PS were assessed as predictors of cure, including cases where operations were extended to excise contralateral positive histology. Additional analyses assessed the sensitivity of imaging for ipsilateral excision confirmed by histology, resulting in a cure. Multivariate binary logistic regression was used to express these relationships as ORs with 95% CIs. Further analysis evaluated US as a predictor of cure while controlling for other factors, including PS status and whether the case was a first operation or a revision.

## Results

The average age of patients was 61.3 ± 12.7 years, and 235 (78.6%) were female. The mean preoperative adjusted serum calcium was 2.8 ± 0.2 mmol/L, and the mean preoperative serum PTH was 19.0 ± 18.0 pmol/L. At six-week postoperative follow-up, the mean adjusted serum calcium was 2.4 ± 0.2 mmol/L, and the mean serum PTH was 6.4 ± 6.9 pmol/L. Among the 297 cases, 275 (92.6%) were first-time parathyroidectomies, and 22 (7.4%) were revision procedures. Histological analysis revealed parathyroid adenoma in 274 (92.3%) patients, parathyroid hyperplasia in 14 (4.7%), and parathyroid carcinoma in four (1.3%). No pathological tissue was found in five (1.7%) cases.

A total of 211 (75.1%) US scans were positive. US demonstrated a sensitivity of 79.4%, a specificity of 64.3%, a PPV of 95.3%, and an NPV of 25.7%. Among US-positive patients, 201 (95.3%) achieved a cure. The OR of cure with positive US status was 6.96 (95% CI: 3.13-15.97, p < 0.0001). PS was positive in 178 (73%) cases, with a sensitivity of 76.2%, specificity of 48.3%, PPV of 91.6%, and NPV of 21.5%. The OR of cure with positive scintigraphy status was 2.98 (95% CI: 1.35-6.60, p = 0.007). Combined preoperative US and scintigraphy returned concordant positive results in 132 (55.2%) cases. The combined modality demonstrated a sensitivity of 59.4%, specificity of 77.8%, PPV of 95.5%, and NPV of 19.6%. Among concordant positive cases, 126 (95.5%) resulted in a cure (Table 1).

**Table 1:** Accuracy parameters for US, scintigraphy, and combined US and scintigraphy NPV, negative predictive value; PPV, positive predictive value; US, ultrasound

Accuracy parameter	US	Scintigraphy	US and scintigraphy
Sensitivity	79.4%	76.2%	59.4%
Specificity	64.3%	48.3%	77.8%
PPV	95.3%	91.6%	95.5%
NPV	25.7%	21.5%	19.6%

The accuracy of laterality in US- and PS-positive cases was also examined. Among US-positive cases, 90.2% were cured by ipsilateral surgical procedures, with a lateralization sensitivity of 80.3%, specificity of 64.3%, PPV of 95.1%, and NPV of 27.3%. For PS-positive cases, 87.8% were cured by ipsilateral surgical procedures, with a lateralization sensitivity of 76.6%, specificity of 52%, PPV of 93%, and NPV of 21%.

Regarding surgical outcomes, 23 (8.4%) first-time operations did not result in cure, compared with seven (31.8%) reoperations. The OR of cure for revision versus first operation was 0.20 (p = 0.001, 95% CI: 0.072-0.528). In the analysis of cure predicted by US while controlling for PS status, the OR of cure in positive versus negative US cases was 5.233 (95% CI: 2.14-12.775, p < 0.0001). In this analysis, PS was not a significant predictor (OR 2.169, 95% CI: 0.904-5.204, p = 0.083). Further analysis of US as a predictor of cure, controlling for PS status and whether the operation was a revision or first operation, showed that only US status was a significant predictor (OR 4.47, p = 0.001, 95% CI: 1.77-11.251). Other variables, including PS status (p = 0.076) and revision status (p = 0.101), were not significant predictors of cure.

## Discussion

Preoperative localization in primary hyperparathyroidism significantly improves the likelihood of surgical cure, reduces the risk of complications, and shortens operative time. US is widely available, relatively safe, radiation-free, and inexpensive. It has demonstrated good but variable sensitivity (50-80%), influenced by operator experience, lesion location, and number of pathological glands, with consistently high specificity (91-96%) [9-11]. PS is also readily available and less operator-dependent. Although it may yield a higher rate of false positives, particularly in the presence of concomitant thyroid pathology [10,11], some studies report higher sensitivity for parathyroid pathology (54-96%) compared with US, while others find no significant differences in sensitivity or specificity between the two modalities [12]. These findings highlight the differences in imaging characteristics: US provides superior anatomical detail when glands are in their normal position, whereas scintigraphy is more effective in locating ectopic glands and provides valuable physiological information [13].

In our study, US and PS returned comparable rates of positive findings (US: 75.1%; PS: 73%) and sensitivity (US: 79.4%; PS: 76.2%). PPVs for lateralization were also comparable (US: 95.1%; PS: 93%). These results align with those of Scattergood et al. (2019), in which both modalities demonstrated similar sensitivity, and a positive finding was a reliable indicator for a minimally invasive surgical approach [13]. Concordant positive imaging from combined US and PS showed lower sensitivity (59.4%) but higher specificity (77.8%) than US alone, with a similar PPV (95.2%). However, multivariate analysis using both US and PS as predictors of cure indicated that only US was a significant predictor, suggesting that US is the more useful primary imaging modality, supporting NICE guideline recommendations [14].

Twenty-two cases were either revised or not cured and remained under surveillance due to multigland disease, ectopic lesions with concomitant thyroid pathology, or lesions missed by both US and PS. In such challenging cases, alternative localization modalities have shown promise. 4D CT, which leverages the variable perfusion of overactive parathyroid glands to differentiate them from surrounding tissue, has demonstrated higher sensitivity and PPV than US and PS, particularly in cases with negative or inconclusive imaging [15-18]. Similarly, 4D SPECT/CT provides detailed anatomical and physiological information, which is valuable in the presence of confounding factors such as multigland disease, thyroid pathology, or obesity. However, both 4D CT and 4D SPECT/CT are associated with higher radiation exposure and increased cost [19,20].

Limitations of this study include its retrospective design and single-institution setting, which limit generalizability and may introduce selection bias. Additionally, US and PS interpretations are operator- and observer-dependent; in some cases, US was performed with prior knowledge of PS-positive findings, which may have increased the observed rate of positive US findings.

## Conclusions

Image-guided focused parathyroidectomy results in superior cure rates in patients with primary hyperparathyroidism. In our practice, US performed by specialists in identifying parathyroid candidate lesions predicted a high rate of all-cause cure and cure when histology was concordant with US findings. We were unable to demonstrate that PS, as an adjunct to US, significantly improves cure rates. Patient groups at high risk of operative failure include those undergoing reoperation, those without positive US or PS findings, and those with concomitant thyroid pathology. In these patients, additional imaging with 4D CT may be considered, as it can delineate candidate lesions in greater detail for preoperative planning.
